# Partial hepatectomy accelerates colorectal metastasis by priming an inflammatory premetastatic niche in the liver

**DOI:** 10.3389/fimmu.2024.1388272

**Published:** 2024-06-10

**Authors:** Jost Luenstedt, Fabian Hoping, Reinhild Feuerstein, Bernhard Mauerer, Christopher Berlin, Julian Rapp, Lisa Marx, Wilfried Reichardt, Dominik von Elverfeldt, Dietrich Alexander Ruess, Dorothea Plundrich, Claudia Laessle, Andreas Jud, Hannes Philipp Neeff, Philipp Anton Holzner, Stefan Fichtner-Feigl, Rebecca Kesselring

**Affiliations:** ^1^ Department of General and Visceral Surgery, University Medical Center Freiburg, Freiburg, Germany; ^2^ Faculty of Medicine, University of Freiburg, Freiburg, Germany; ^3^ German Cancer Consortium (DKTK) Partner Site Freiburg, Freiburg, Germany; ^4^ German Cancer Research Center (DKFZ), Heidelberg, Germany; ^5^ Eye Center, University Medical Center Freiburg, Freiburg, Germany; ^6^ Department of Medicine I, University Medical Center Freiburg, Freiburg, Germany; ^7^ Division of Medical Physics, Department of Diagnostic and Interventional Radiology, University Medical Center Freiburg, Faculty of Medicine, University of Freiburg, Freiburg, Germany

**Keywords:** colorectal cancer, liver metastasis, partial hepatectomy, premetastatic niche, tight junctions

## Abstract

**Background:**

Resection of colorectal liver metastasis is the standard of care for patients with Stage IV CRC. Despite undoubtedly improving the overall survival of patients, pHx for colorectal liver metastasis frequently leads to disease recurrence. The contribution of this procedure to metastatic colorectal cancer at a molecular level is poorly understood. We designed a mouse model of orthograde metastatic colorectal cancer (CRC) to investigate the effect of partial hepatectomy (pHx) on tumor progression.

**Methods:**

CRC organoids were implanted into the cecal walls of wild type mice, and animals were screened for liver metastasis. At the time of metastasis, 1/3 partial hepatectomy was performed and the tumor burden was assessed longitudinally using MRI. After euthanasia, different tissues were analyzed for immunological and transcriptional changes using FACS, qPCR, RNA sequencing, and immunohistochemistry.

**Results:**

Mice that underwent pHx presented significant liver hypertrophy and an increased overall metastatic load compared with SHAM operated mice in MRI. Elevation in the metastatic volume was defined by an increase in *de novo* liver metastasis without any effect on the growth of each metastasis. Concordantly, the livers of pHx mice were characterized by neutrophil and bacterial infiltration, inflammatory response, extracellular remodeling, and an increased abundance of tight junctions, resulting in the formation of a premetastatic niche, thus facilitating metastatic seeding.

**Conclusions:**

Regenerative pathways following pHx accelerate colorectal metastasis to the liver by priming a premetastatic niche.

## Graphical Abstract

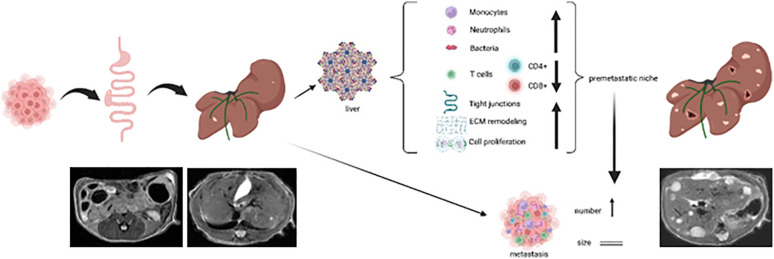

## 1 Introduction

Colorectal cancer (CRC) ranks second among the cancer-associated deaths worldwide (1). While early CRC is associated with a rather good prognosis after tumor resection, distant metastasis is an interdisciplinary challenge and reduces 5-year-survival (5-ys) to only 11% (2). The liver is the most frequent and clinically relevant site of metastasis. In cases of isolated liver metastasis (LM), complete resection of hepatic metastases in combination with adjuvant chemotherapy can raise the 5-ys to 35.5%, with even long-term survivors (3). These results have led to more rigorous surgical approaches in resecting LM, even though complete (R0) resection shows a relapse of disease in up to 60% of patients, with the liver as the most probable site of recurrence (4, 5). Along with the regenerative capacity of the liver, this leads to an increasing number of patients with stage IV CRC undergoing repetitive surgical liver metastasectomies (6). The regeneration of the liver after partial hepatectomy (pHx) and the underlying molecular mechanisms have been studied extensively, revealing that IL-6 (7) and multiple growth factors (TNFα, TGFβ, EGF and VEGF) are key regulators of regeneration and proliferation in the residual liver (8, 9). All of these, as well as many downstream targets (e.g. β-Catenin, Stat3), have been linked to a generally unfavorable prognosis in cancer patients. IL-6 and the above-mentioned growth factors have also been described in the context of the premetastatic niche, a concept that describes tissue alterations facilitating the engraftment of circulating tumor cells in the target organ, thus leading to successful implantation of metastases (10, 11). This raises the question of the effects of pHx on metastatic disease, which has been addressed in several animal studies. The published studies rely on animal models of CRC liver metastasis, in which hematogenous seeding is achieved via portal vein or splenic injection. Therefore, these models are rather artificial and exclude the crucial influence of the microenvironment of the primary tumor on CRC metastasis as well as the significant changes that cancer cells undergo before entering the bloodstream (e.g. epithelial-mesenchymal transition (EMT)) (12–15). As the liver is often the tumor manifestation defining the patients’s prognosis, in several cases of synchronous metastasis of CRC, resection of LM is performed prior to the resection of the primary tumor. This liver-first approach urges clinicians to understand the effects of pHx in the presence of the primary tumor. To analyze the missing aspects, we developed a reproducible mouse model of orthograde colorectal liver metastasis and characterized the impact of partial hepatectomy on the immunological compartments in primary CRC, LM and healthy liver, and on tumor progression in depth.

## 2 Methods

### 2.1 Organoid culture

APTAK organoids were kindly gifted by Florian Greten, Georg Speyer Haus, Frankfurt. These cells are murine colorectal carcinoma cells holding the following mutational features: Deletions in the *Apc*-, *T*r*p53*- and *Tgfbr2*-gene, a myristoylated Akt-1 with constitutively activated signaling and a *Kras* ^G12D^-mutation (16). The APTAK organoids were maintained in Advanced DMEM-F12 (Gibco/Thermo Fisher Scientific, Waltham, MA, USA) supplemented with penicillin (100 U/ml)/streptomycin (100 µg/ml) (Gibco/Thermo Fisher Scientific), HEPES 10 mmol/L (Invitrogen/Thermo Fisher Scientific), Glutamax 2 mM (Invitrogen/Thermo Fisher Scientific), N2 1× (Gibco/Thermo Fisher Scientific), B27 1× (Gibco/Thermo Fisher Scientific), and N-acetylcysteine 1 mmol/L (Sigma-Aldrich, St. Louis, MO, USA) and supplemented with hygromycin (200 µg/ml, Invitrogen/Thermo Fisher Scientific) and puromycin (2 µg/ml, InvivoGen, San Diego, CA, USA) (hereafter referred to as basal medium). The organoids were split every 3–5 days at a 1:10 ratio and cryoconserved for long-term storage.

### 2.2 Animal studies

All animal studies were performed using 8–12 weeks old C57Bl6/J-mice (purchased from Charles River, Wilmington, MA, USA) at the pathogen-free barrier facility of the CEMT, University Medical Center Freiburg. The experiments were planned and carried out in accordance with the institutional guidelines and all experiments were approved by the regional board (Regierungspräsidium Freiburg, G21/001).

### 2.3 Orthotopic organoid transplantation mouse model

We adapted the subserosal orthotopic organoid implantation technique described by Fumagalli et al. (17). APTAK organoids cultured in Cultrex RGF basement membrane extract (BME) Type 2 (R&D Systems, Minneapolis, MN, USA) were dissociated into single cells by mechanical disruption and washed with cold PBS. After determining the cell number with the cell counter TC20 (BioRad, Hercules, CA, USA), the appropriate cell number was resuspended in collagen I/5x neutralization buffer (vol/vol-ratio 1:4), and collagen domes of 100.000 cells/10 µl were plated on a 6-well plate. After 30 min polymerization at 37°C, basal medium was added and cells were incubated over night until implantation. Mice analgesia was performed with intraperitoneal (i.p.) injection of 0.1 mg/kg bodyweight (BW) buprenorphine (Temgesic, Schering-Plough, Kenilworth, NJ, USA). Anesthesia was induced by 2.5 Vol% Isoflurane (Isoflurane Piramal, Piramal, Mumbai, India) with the SomnoSuite^®^ Low-Flow Anesthesia System (Kent Scientific Corporation, Torrington, CT, USA). The unconscious mice were positioned on a heating pad with all legs fixed and the abdomen was shaved while anesthesia was maintained by continuous application of isoflurane via mouthpiece. After disinfection of the skin, a median laparotomy of approximately 1 cm was performed on the lower abdomen. The cecum was mobilized using two cotton swabs and placed on a wet gauze. Under the microscope, a 2 mm incision of the serosal layer was made and a deep subserosal pocket was created in which the organoid-collagen-graft was fully placed. After covering the graft with the serosal layer, an anti-adhesion layer (Seprafilm, Baxter, Deerfield, IL, USA) was placed on top. The cecum was then replaced in the abdominal cavity and the abdominal wall and skin were closed in two layers using 6–0 Vicryl (Ethicon/Johnson&Johnson, New Brunswick, NJ, USA) suture. The duration of the surgical procedure was approximately 10–15 minutes.

### 2.4 Magnetic resonance imaging

MR imaging was performed using a 9.4 tesla small bore animal scanner (BioSpec 94/21, Bruker Biospin, Karlsruhe Germany) and a dedicated mouse quadrature-resonator (Bruker). The mice were anesthetized under spontaneous breathing conditions using Isoflurane. Heart and respiration rates were continuously monitored and maintained at a constant level. Gating was used to reduce motion and blood flow artifacts during the scan. The MRI protocol consisted of a localizer and a T2-weighted spin echo RARE (Rapid Acquisition with Relaxation Enhancement) sequence. This sequence was performed to delineate the tumor and eventual metastasis from the surrounding healthy tissue. The RARE sequence in axial orientation featured a field-of-view (FOV) of 28 mm (1), a matrix size of 280 x 280 pixels, and an in-plane resolution of 100 x 100 µm (1). The slice thickness was 0.75 mm (TR/TEeff/FA: 300 ms/36 ms/180°). The number of slices was adjusted to the measured volume (on average 30) to ensure complete coverage of the abdomen. The Bruker ParaVision 6.0.1 software was used for imaging and images were analyzed using the NORA software supplied by the Medical Center of the University of Freiburg. The volumes of all hepatic lobes and metastases were assessed individually by manually marking the respective lobes/LMs as regions of interest (ROIs) in each section.

### 2.5 Ultrasound

During isoflurane anesthesia mice were placed on a heating pad and the abdomen was shaved. Prewarmed ultrasound gel was applied to the abdomen and a VEVO 3100 (Fujifilm/VisualSonics, Toronto, Canada) ultrasound together with a small animal transducer was used for ultrasound imaging.

### 2.6 Partial hepatectomy

For distribution among the control-, SHAM-, and pHx-groups, we assessed the preoperative hepatic tumor load using MRI. Animals displaying an approximately similar number and volume of LM in the imaging were allocated equally among the different protocols to reduce the influence of cage-specific phenotypes. After general analgesia and anesthesia with buprenorphine and isoflurane as described above, mice were shaved and placed on a heating pad. After disinfection, a median laparotomy of approximately 2 cm, starting at the xiphoid, was performed and retractors were placed on both sides of the abdominal wall and under the xiphoid. The falciforme ligament and the membrane between the medial and left hepatic lobes were dissected. A 3–0 Vicryl (Ethicon/Johnson&Johnson) suture was placed around the base of the medial lobe and tightened with 3 knots, after which the ischemic lobe was cut approximately 0.1 cm distal to the suture (**Supplementary Figure 1A**). The resected lobe was placed in cooled PBS Buffer on ice until further analysis. The abdominal cavity was flushed with prewarmed saline, and the abdominal wall and skin were closed using 6–0 Vicryl (Ethicon/Johnson&Johnson) stitches. The pHx procedure took approximately 15 min. SHAM mice underwent laparotomy, including dissection of the falciforme ligament and the membrane between the medial and left lobes without resection of the lobe. Control animals received general anesthesia without any surgical procedures.

### 2.7 Tumor dissociation

Animals were euthanized by cervical dislocation. Subsequently, the abdomen was opened and each lobe of the liver was resected directly at the vena cava. The lobes were weighed and all visible liver metastases were dissected while the organ was sliced. The entire cecum was resected and the tumor tissue was separated from the healthy intestine. We collected samples for RNA isolation (stored in RNAprotect buffer (Qiagen, Netherlands)) and for protein isolation from all tissues mentioned. Healthy liver tissue was minced and dissociated using the Liver Dissociation Kit, mouse (Miltenyi Biotec, Bergisch Gladbach, Germany) according to the manufacturer’s protocol. The primary tumor and LM were minced into <1 mm pieces and dissected using the Tumor Dissociation Kit, mouse (Miltenyi Biotec) according to the manufacturer’s instructions. The single cell suspension was filtered through a 100 µm cell strainer and ACK lysis buffer (Gibco/Thermo Fisher Scientific) was added to lyse erythrocytes during a 1.5 minutes incubation.

### 2.8 Flow cytometry

Cells were transferred to 100 µl FACS buffer (PBS + 0,5% BSA), incubated with TruStain FcX PLUS (anti-mouse CD16/32) antibody (BioLegend, San Diego, CA, USA) for 15 min and then stained with the respective fluorescent antibodies for 60 min. Cells were then washed with FACS buffer (for extracellular staining) and fixed with 1x Fixation/Permeabilization buffer for 60 min. For intracellular staining, we used the eBioscience™ Foxp3/Transcription Factor Staining Buffer Set (Invitrogen/Thermo Fisher Scientific) according to the manufacturer’s instructions. All FACS antibodies used were supplied by BioLegend and used undiluted with 1 µl/100 µl cell suspension. FACS was performed using Beckman Coulter Gallios (Beckman Coulter, Brea, CA, USA) or BD LSRFortessa (Becton Dickinson, Franklin Lakes, NJ, USA). Data were analyzed using Kaluza Software (Beckman Coulter). Cellular aggregates were excluded using SSC-W.

### 2.9 T cell stimulation

Equal amounts of single cells from the tumor/liver dissociation were seeded in RPMI-Medium (+ 1% Pen/Strep + 1% HEPES + 5 µl beta-mercaptoethanol) on a 12-well plate overnight. The cells were then stimulated by adding Cell Activation Cocktail (BioLegend) for 3 hours, followed by addition of Brefeldin A (BioLegend) for 2 hours. The cells were then harvested and stained for FACS analysis, as described above.

### 2.10 RNA isolation and cDNA synthesis

Tissue samples for RNA isolation were stored in RNAprotect Buffer. The tissue was shredded in 350 µl RLT buffer (Qiagen, Venlo, Netherlands) + 1% beta-mercaptoethanol. Further RNA isolation was carried out using the RNeasy Mini Kit (Qiagen) according to the manufacturer’s instructions. RNA concentrations were measured using a NanoDrop™ spectrophotometer (Thermo Fisher Scientific). For cDNA synthesis RNA was transcribed using ProtoScript II First Strand cDNA Synthesis Kit (New England Biolabs, Ipswich, MA USA) according to the manufacturer’s instructions. cDNA was finally diluted to 3 ng/µl for qPCR experiments.

### 2.11 DNA isolation

For quantification of bacterial DNA, we isolated DNA from tissue samples using the DNeasy Blood & Tissue Kit (Qiagen) according to the manufacturer’s instructions, and concentrations were assessed using a NanoDrop™ spectrophotometer (Thermo Fisher Scientific).

### 2.12 Quantitative real-time PCR

Quantitative Real-Time PCR was performed using QuantiTect SYBR Green RT PCR mastermix (Qiagen) with 7.5 ng template cDNA and 5 pmol of each primer (purchased from Eurofins Genomics, Ebersberg, Germany; primer sequences supplied the supplements). qPCR was carried out on a Roche LightCycler 480 (Roche, Basel, Switzerland) with each sample-gene combination in triplicates. The protocol consisted of a 10 min activation step at 95°C followed by 50 cycles of 45 s amplification at 60°C and inactivation at 95°C for 15 s. 18S was used as a housekeeping gene, Cp values were calculated using the LightCycler480 software (Roche), and the relative expression of target genes was calculated by comparative method after normalization to 18S-expression.

### 2.13 Bacterial 16S qPCR

In order to quantify bacterial infiltration, we performed qPCR with amplification of the bacterial V6 region using five different primers (see Supplements). Each sample was measured in triplicates using the QuantiFast SYBR^®^ Green PCR Kit (Qiagen). 100 ng DNA was mixed with 500 nM primers. By diluting the DNA of *Bifidobacterium actinocoloniiform*e (DSMZ-Deutsche Sammlung von Mikroorganismen und Zellkulturen GmbH, DSM22766) in 10-fold series we generated a standard curve using the Threshold cycle (Ct) values. To quantify the sample bacterial DNA, we correlated the average Ct-values to the standard curve of log values from *the Bifidobacterium actinocoloniforme*.

### 2.14 Bulk RNA sequencing

RNA sequencing was performed at Genewiz, Germany, using the standard Illumina protocol to create raw sequence files (.fastq files). Poly(A) mRNA was used to prepare cDNA libraries, which were then sequenced as 2x 150 bp paired end using the Illumina NovaSeq instrument (Illumina, San Diego, CA, USA).

### 2.15 RNA seq analysis

Raw sequencing files were uploaded to the galaxy web platform (https://usegalaxy.eu) (18) and quality was assessed using FASTQC. Owing to good quality and no adaptor content, trimming was not necessary. Data were mapped to the murine reference genome (GRCm39) provided by GENCODE (www.gencodegenes.org, downloaded September 2021) using the STAR aligner (19) in standard settings for paired sequencing data. Reads were assigned to counts using featureCounts software (20) and the gene annotations also provided by GENCODEs (downloaded September 2021). Paired-end settings were used for featureCounts software, and a quality score of >10 was required for counting. Data were imported in R 4.0.2 (https://www.r-project.org), and differentially expressed gene analyses were conducted using DESeq2 (21). A batch effect was removed using the limma::removeBatchEffect() function (22). Differentially expressed genes were defined as padj <0.05 and abs(log2 FC) >1. The Ensembl gene ID was converted to MGI symbols using the biomaRt package (23). The fgsea package (24) conducted Gene Set Enrichment Analysis (GSEA) by ranking all genes with at least >10 counts according to the log2 FC, which were previously shrunken by the apeglm logarithm (25) before. Gene sets were accessed from MsigDB (26) and downloaded on the 26^th^ of May 2023. For visualization, ggplot2 (27), ggraph and ComplexHeatmaps (28) were used.

### 2.16 Protein isolation

Tissue samples were shredded in RIPA buffer containing phosphatase and proteinase inhibitors (Phosstop (Roche), protease inhibitor cocktail, sodium orthovanadate, and PMSF (Santa Cruz, Santa Cruz, CA, USA) on ice. Three cycles of freezing and thawing were applied using liquid nitrogen, and the suspension was centrifuged at maximum speed for 10 min. The supernatant containing the protein lysate was transferred to a new tube. Protein concentration was measured using a Pierce BCA-Kit (Thermo Fisher Scientific) and an Azure Ao Microplate Reader (Azure Biosystems, Dublin, CA; USA).

### 2.17 Western blot

Protein samples were aliquoted with 4x Laemmli Buffer (BioRad) and denatured at 95°C for 3 min. An equal amount of protein was loaded on a 12–20% Mini-Protean TGX precast gel (BioRad) together with a color-coded prestained broad range protein ladder (Cell Signaling Technology, Danvers, MA, USA). After SDS-electrophoresis, the proteins were blotted onto a PVDF membrane using the trans-blot turbo transfer system (BioRad) according to the manufacturer’s instructions. The membrane was then blocked with 5% BSA/TBST for 60 min at RT and incubated with the respective primary (Anti-S100A8, Anti-MMP9, Anti-Claudin-2, Anti-Occludin, and Anti-ZO-1) and secondary (anti-rabbit IgG) antibodies according to the manufacturer’s instructions (a detailed description of the antibodies used is supplied in the Supplements). For detection, the Immobilon Crescendo Western HRP substrate (Merck Millipore, Burlington, MA, USA) was added to the membrane and imaging was carried out using a ChemiDoc BioRad imager (BioRad).

### 2.18 Immunohistochemistry

Tissue samples were fixed in 4% PFA for 24 hours, dehydrated and embedded in paraffin. Sections of 3 µm were cut, rehydrated, and heat induced epitope retrieval was carried out with citrate or TE-Buffer (Zytomed Systems, Berlin, Germany). Sections were then blocked with 2.5% BSA and avidin-biotin-block (Zytomed Systems) according to the manufacturer’s instructions. Sections were then incubated with the primary antibody overnight and biotinylated secondary antibody for 1 h according to the manufacturer’s instructions (see a detailed description of the antibodies used in Supplements). Positive staining was visualized using DAB (Zytomed Systems), and the sections were counterstained with hematoxylin before dehydration and mounting with ROTI^®^ Histokitt (Carl Roth, Karlsruhe, Germany). Slides were digitalized using a Zeiss Axio Scan Z.1 Microscope Slide Scanner (Zeiss, Jena, Germany).

### 2.19 Immunofluorescence

Slides were treated until secondary antibody incubation as described in the *Immunohistochemistry* section, and then incubated with the Vector^®^ TrueVIEW^®^ Autofluorescence Quenching Kit (Biozol, Eching, Germany) to reduce autofluorescence. After washing, nuclear staining was performed using NucBlue™ Fixed Cell Stain ReadyProbes™ (Thermo Fisher Scientific). Sections were then mounted with VECTASHIELD Vibrance Antifade Mounting Medium (Biozol) and scanned using a Zeiss Axio Scan Z.1 Microscope Slide Scanner (Zeiss).

### 2.20 Statistics

For statistical analysis, we used a one-way analysis of variance (ANOVA) test with Tukey’s *post-hoc* analysis when comparing multiple samples or a two-tailed Students-t-test for two variables. Statistical significance was set at p-values <0.05, and significance levels are represented in the graphic as follows: *** = p<0.001, ** = p<0.01, * = p<0.05, n.s. = not significant. All statistical analyses were performed using GraphPad Prism.

## 3 Results

### 3.1 Orthograde CRC metastasis mouse model - implantation of APTAK organoids frequently leads to orthograde liver metastases of CRC

**Figure 1:**
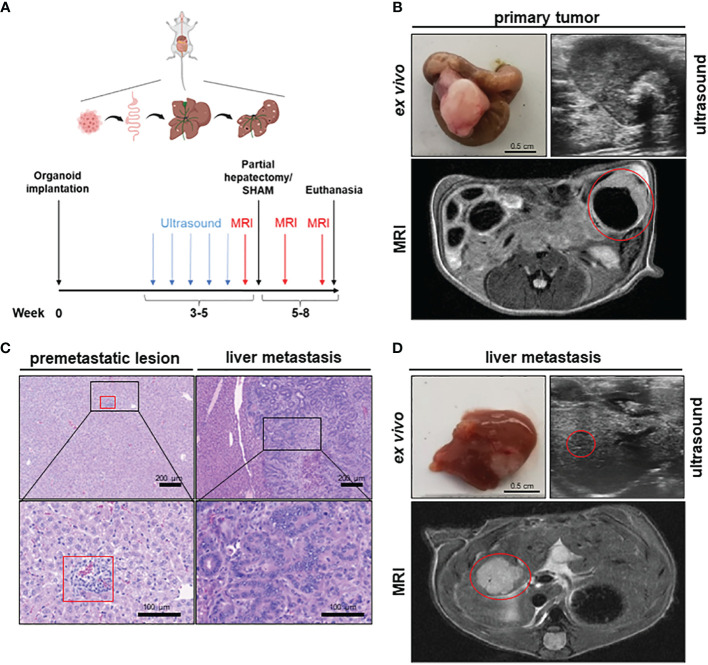
A novel mouse model to study the effects of partial hepatectomy in orthotopic metastasized colorectal carcinoma. **(A)** Overview of experimental layout (top) and timeline for procedures (bottom); **(B)** Representative images of primary tumor ex vivo (scale bar = 0.5 cm) and T2-weighted transversal MRI of early primary tumor (red circle) and locally advanced primary tumor in ultrasound; **(C)** Representative HE-staining of liver with premetastatic lesion (red square) and of advanced liver metastasis (LM) (scale bars in left panels = 200 µm and in right panels =100 µm); **(D)** Representative images of liver and LM ex vivo (scale bar = 0.5 cm) and of T2-hyperintense LM (red circle) in the medial hepatic lobe (max. diameter 7 mm) in transversal T2-weighted MRI and in ultrasound of the same liver metastasis (red circle).

To establish a mouse model of orthograde CRC metastasis we used murine CRC tumor organoids deficient for *Apc*, *Trp53* and *Tgfbr2* and that display a *Kras* gain of function mutation (G12D mutant) together with a constitutively activated Akt1 (hereafter referred to as APTAK organoids) (29). We combined this APTAK mouse model with a partial hepatectomy model to monitor the effect of pHx on the background of orthograde colorectal liver metastasis (**Figure 1A**). APTAK organoids were surgically implanted under the serosal layer of the distal cecum, leading to carcinomas with luminal contact after 2 weeks (**Figures 1B–D**). These tumors caused cellular aggregates that appeared in the liver after 3–4 weeks (microscopically detectable in hematoxylin/eosin (HE)-sections) (**Figure 1C**) and macroscopically visible metastases after 5–6 weeks (**Figures 1C, D**). To further characterize the early-appearing cellular accumulations in the liver, we performed an immunohistochemistry (IHC) of liver sections. While the liver metastases and primary tumors displayed plenty of EpCam-positive cells, we did not find any EpCam-positive epithelial cells in the microscopically visible perivascular cellular aggregates (**Supplementary Figure 1A**). Hence, we adapted the term premetastatic lesions.

To objectify the metastatic load in the liver, magnetic resonance imaging (MRI) was performed prior to liver resection. Detectable liver metastases in MRI appeared after 4–5 weeks as T2-hypointense lesions (**Figure 1D**), with the smallest distinguishable measuring 0.2 mm in diameter. To measure the time to metastasis and general tumor burden, mice were screened weekly for detectable liver metastases with ultrasound starting 3 weeks after implantation. In comparison to MRI, detection of LM in ultrasound was delayed with visible lesions starting at >1 mm in diameter (**Figure 1D**). After resection of the medial lobe (**Supplementary Figure 1B**), mice were screened again for metastases on postoperative day (POD) 7 and 14 in MRI. The animals were then euthanized and their organs were harvested for further analysis. Therefore, we set up a valuable orthograde CRLM mouse model suitable for analyzing pHx, which resembles the human situation.

### 3.2 Medial lobe resection induces significant hypertrophy of the residual liver

**Figure 2:**
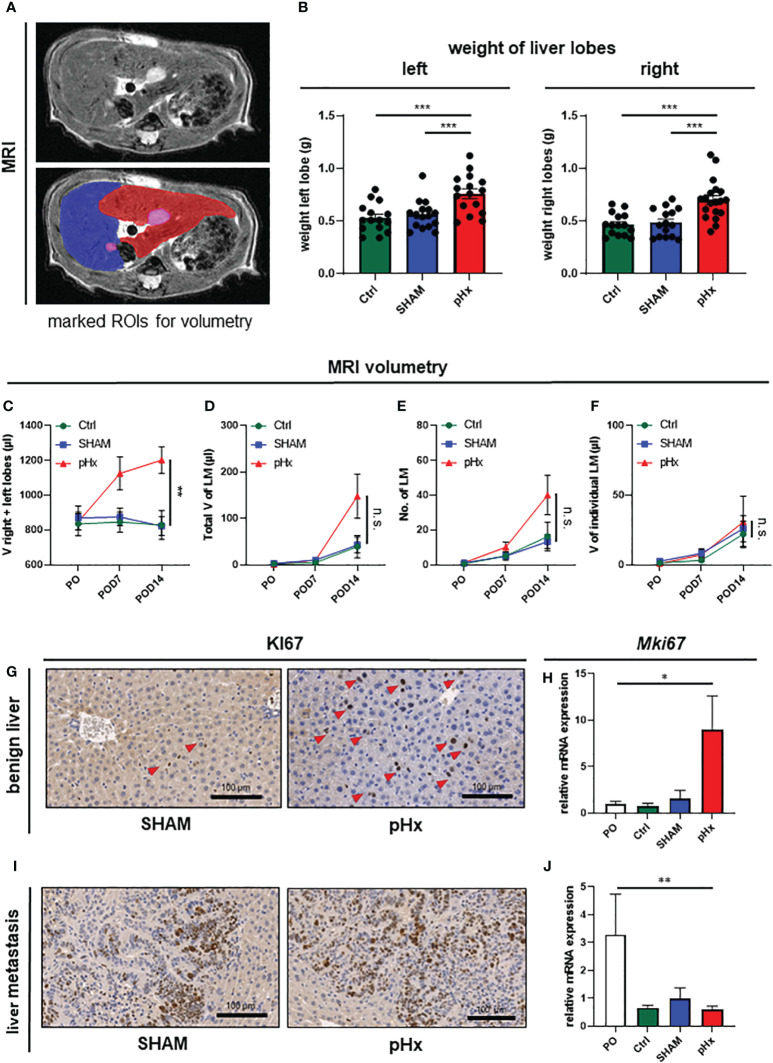
Medial lobe hepatectomy induces hypertrophy of the residual liver and increases overall metastatic volume and number without affecting the individual growth of each metastasis. **(A)** Representative MRI of liver and LM following pHx (POD7) in native T2-weighted MRI (top) and with marked regions of interest (ROI) (bottom): blue: right upper hepatic lobe, red: left hepatic lobe, pink: LM; **(B)** Boxplots showing the weight in grams of left and right liver lobes after euthanasia of Ctrl group (n=15/15), SHAM group (n=16/15) and pHx group (n=16/19); **(C)** Time course analysis of the combined volume of healthy left and right lobes in µl measured in MRI of Ctrl (n=5), SHAM (n=6) and pHx (n=9) group; **(D)** Time course analysis of total volume of liver metastases in left and right lobes in µl measured in MRI for Ctrl (n=7), SHAM (n=15) and pHx (n=19) group; **(E)** Time course analysis of the number of LM in left and right lobes assessed in MRI for Ctrl (n=7)), SHAM (n=14) and pHx (n=15) group; **(F)** Time course analysis of the individual growth in volume of LM in MRI for Ctrl (n=7), SHAM (n=14) and pHx (n=12) group; **(G)** Representative immunohistochemistry of Ki67 of healthy liver following SHAM- and pHx-surgery. Nuclear counterstaining was performed with hematoxylin, red arrows point to Ki67^+^ cells; **(H)** Boxplot representing the relative expression of *MKi67* in healthy liver tissue analyzed by qPCR in PO (n=14), Ctrl (n=4), SHAM (n=10) and pHx (n=12) group; **(I)** Immunohistochemistry of Ki67 of liver metastasis. Nuclear counterstaining was performed with hematoxylin; **(J)** Boxplot representing the relative expression of *Mki67* in liver metastasis analyzed by qPCR for PO (n=5), Ctrl (n=3), SHAM (n=8) and pHx (n=14) group. (Scale bars = 100 µm, bar plots represent mean ± standard error of the mean (SEM), p-values calculated via one-way ANOVA Tukey test, * = p<0.05, **, p <= 0.01 *** = p<0.001).

In order to verify the hypertrophic effect on the residual liver and to evaluate the effect of pHx on the metastatic load we performed volumetric analysis of the MR images prior to surgery and 7 and 14 days postoperatively (**Figure 2A**). Following pHx, the mice developed significant hypertrophy of ~50% of the residual right and left lobes compared to control and SHAM-operated mice both in weight (**Figure 2B**) and volume measured in MRI (each p<0.001) (**Figure 2C**, **Supplementary Figure 1C**). The hypertrophy was already seen in MRI on POD7 but further increased until POD14. The total volume of healthy liver tissue in pHx mice on POD14 was comparable to that in both control groups (**Supplementary Figure 1D**). We could conclude that 1/3 partial hepatectomy in our model is sufficient to generate significant hypertrophy, consistent with the effects of a major hepatectomy in humans.

### 3.3 Partial hepatectomy increases *de-novo* metastasis without affecting growth of present metastasis

As pHx has been linked to a potentially prometastatic effect in injection models of colorectal liver metastasis before (12–14), we were curious about the impact of resection in our model of orthograde metastasis. Regarding the metastatic burden in the animals, we found no difference in the total metastatic volume within the liver on POD7 between control, SHAM, and pHx mice. On POD14, on the other hand, an increase in the metastatic volume in the residual right and left lobes was observed in the hepatectomy group compared to that in both control groups (**Figure 2D**, **Supplementary Figure 1E**). The same effect was observed when the metastases in the medial lobe of the control groups were included in the analysis (**Supplementary Figure 1G**). The increase in total metastatic volume was explained by an increase in the total number of metastases rather than by accelerated growth of the present metastases, and the number of metastases was elevated after pHx compared to the two other groups, analogous to the total metastatic volume (**Figure 2E**, **Supplementary Figure 1F**). Regarding the size of each liver metastasis individually over the postoperative period, we found no difference in the volume increase between pHx, SHAM, and control mice (**Figure 2F**). Consistently, at transcriptional level, we found a significant induction of cell proliferation represented by *Ki67*-expression within the healthy liver after pHx compared to SHAM and control mice, both in quantitative real-time polymerase chain reaction (qPCR) and immunohistochemistry (IHC) with p<0.05 (**Figures 2G, H**). However, proliferation in liver metastases was not altered following hepatectomy (**Figures 2I, J**). The detected general decrease in *Ki67*-expression in all three models (**Figure 2J**) compared to preoperative levels can be explained by the increase in necrotic tissue in larger metastases over time. In summary, pHx increased the number of *de novo* metastases without affecting the proliferation and growth of each metastasis. Therefore, we assumed that one of the major implications of pHx is priming of the liver for *de novo* metastatic implantation.

### 3.4 Partial hepatectomy induces an inflammatory phenotype in liver and LM

**Figure 3:**
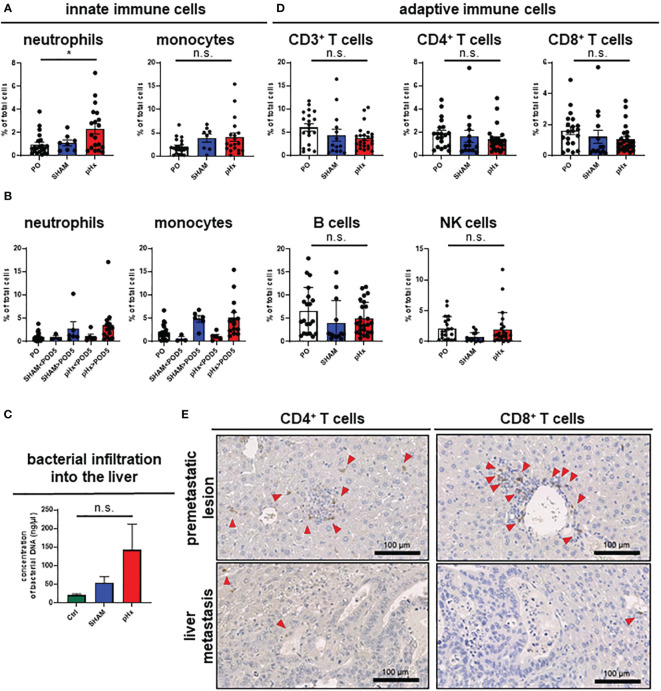
pHx induces neutrophil accumulation and increases bacterial abundance in the residual liver. **(A)** Flow cytometric analysis with staining of different populations of innate immune cells in healthy liver tissue (neutrophils: CD45+/CD11b+/Ly6G+/CD64-, monocytes: CD45^+^/CD11b^+^/Ly6G-/CD64^+^) for PO (n=21), SHAM (n=8) and pHx (n=19) group, **(B)** Time course of infiltration of the liver with innate immune cells before POD5 (range POD3–5) and after POD5 (range POD7–14) for PO (n=21), SHAM<POD5 (n=3), SHAM>POD5 (n=6), pHx<POD5 (n=5) and pHx>POD5 (n=14) group; **(C)** Concentration (ng/µl) of bacterial DNA in healthy liver measured with qPCR for Ctrl (n=5), SHAM (n=5) and pHx (n=7) group; **(D)** FACS analysis of different populations of adaptive immune cells in healthy liver tissue following pHx (NK cells: CD45^+^/CD3^-^/NK1.1^+^, T cells: CD45^+^/CD3^+^/NK1.1^-^, B cells: CD45^+^/CD3^-^/B220^+^) for PO (n=20), SHAM (n=14) and pHx (n=25) group; **(E)** Representative immunohistochemistry of the distribution of CD4^+^ and CD8^+^ T cells in healthy liver and accumulation in premetastatic lesions (top) with absence in macroscopic metastasis (bottom). Red arrows point to the cells of interest. (Scale bars = 100 µm, bar plots represent mean ± SEM). n.s. non significant.

It is well established that inflammation and the tumor microenvironment (TME) heavily influence CRC carcinogenesis and metastasis (30), therefore we analyzed the effect of pHX on the TME composition of primary tumors (PT) and liver metastases (LM), and immune cell compartments in healthy liver tissues. After generating single-cell suspensions of all three tissues, immune cell compositions were analyzed using fluorescence-activated cell sorting (FACS). Regarding innate immune cells, we found an increased number of neutrophils both in healthy livers and metastases after pHx, as well as a moderate increase in monocyte count (**Figure 3A**, **Supplementary Figure 2A**). Comparing the abundance of these cell types at different time points after resection revealed that the accumulation in the liver did not occur before POD5 (**Figure 3B**). These changes were not accompanied by a general increase in neutrophil or monocyte abundance in the peripheral blood (**Supplementary Figure 2A**). As surgical stress has been linked to an increase in myeloid-derived suppressor cells (MDSC) in tumors, we characterized the MDSC subsets in the liver and peripheral blood in depth. We did not observe a significant difference in the abundance of both polymorphonuclear (PMN-MDSCs) and monocytic MDSCs (M-MDSCs) in either tissue after pHx. However, a change in the PMN-MDSC subtypes towards a monocytic phenotype [described by Veglia et al. (31)] was observed: pHx caused an increase in tumor-associated CD14^high^ PMN-MDSCs in the liver, while decreasing CD14^low^ cells (**Supplementary Figure 2B**). As the microbiome is an important constituent of the TME of CRC and affects CRC pathogenesis (30), we analyzed bacterial infiltration in the liver after pHx. Along with the increased infiltration of myeloid cells, we found an increased presence of bacteria within the liver following pHx (**Figure 3C**) as analyzed by qPCR, whereas bacterial infiltration of the LM was not altered by pHx (**Supplementary Figure 2C**). Taken together, pHx induces an inflammatory composition with an elevated abundance of innate immune cells in the residual liver as a delayed effect, which may be supported by increased bacterial infiltration.

### 3.5 pHx slightly abates T cell abundance with no effect on T cell exhaustion, cytotoxicity, and activation

Having shown that pHx surgery alters innate immune cell responses, we wondered whether pHx also affects adaptive immunity in CRLM. In our pHx mouse model, we could show that the numbers of cells of the adaptive immune system (T cells, B cells and NK cells) in the liver or LM did not change significantly after the respective surgery. However, a trend towards a decrease in CD4^+^ and CD8^+^ T cell abundance in the liver (**Figure 3D**) and especially in metastases (**Supplementary Figure 2D**) following pHx was observed in FACS analysis. To further investigate the role of T cells in this metastasis model, we performed IHC staining for CD4^+^ and CD8^+^ T cells in liver sections. We found that both CD4^+^ and CD8^+^ T cells were scarce in healthy liver tissue, accumulated around premetastatic lesions, and were completely depleted in macrometastases (**Figure 3E**). However, not only the absolute abundance of adaptive immune cells impacts tumorigenesis and metastasis, but also the function of the adaptive immune cells heavily influences tumor progression. Therefore, we functionally characterized the CD8^+^ T cells for their functionality in depth. First, we analyzed T cell cytotoxicity, exhaustion, and activation of CD8^+^ T cells in the liver in the different intervention groups using FACS. While we observed a general increase in exhaustion markers (PD-1, Tcf1) on CD8^+^ T cells along tumor progression in accordance with the present literature, pHx did not affect the expression of exhaustion markers compared to SHAM or control samples (**Supplementary Figure 2E**). Furthermore, we do not detect any changes in T cell cytotoxicity (granzyme B) and activation (CD38) after pHx (**Supplementary Figure 2E**).

### 3.6 pHx induces a prometastatic transcriptomic program including cell cycle progression, epithelial-mesenchymal transition, angiogenesis and hypoxia in the liver

**Figure 4:**
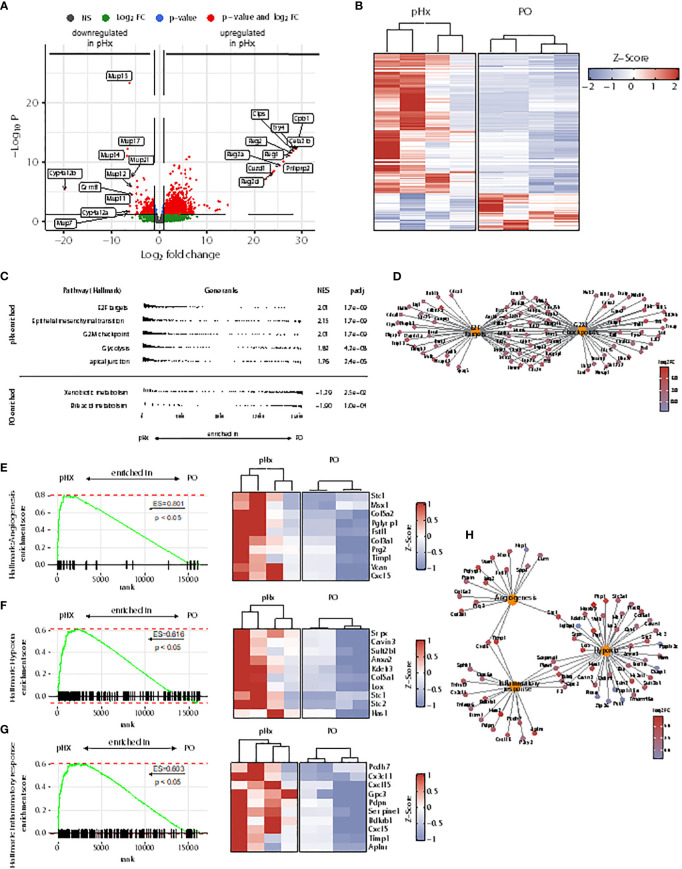
Partial hepatectomy is associated with upregulation of gene sets associated with metastasis in the liver. **(A)** Volcano plot of differently expressed genes (DEGs) in RNA sequencing from healthy liver tissue of 4 pHx and 4 PO samples. The 10 DEGs with the highest or lowest log2 fold change were labeled; **(B)** Heatmap from all DEGs with a base count mean of > 10 visualized by Z-scores; **(C)** Overview of 5 most enriched and 2 most depleted hallmark terms in gene set analysis (GSEA) following pHx; **(D)** Cnet plot visualizing all DEGs related to hallmarks E2F targets and G2M checkpoint and their overlap; **(E-G)** GSEA for hallmarks angiogenesis **(E)**, hypoxia **(F)** and inflammatory response **(G)** and heatmap of the respective 10 leading edge genes (right) visualized by Z-scores; **(H)** Cnet plot of DEGs related to hallmarks angiogenesis, hypoxia, and inflammatory response.

As the livers of mice undergoing pHx shows veritable changes in the immune cell compartments, we wondered whether these changes lead to an impairment of the initiation of the metastatic process in the livers. To analyze the underlying transcriptional alterations in the healthy livers, we performed bulk RNA sequencing from healthy livers of four PO- and four pHx-mice. In total, 1083 upregulated and 289 downregulated differentially expressed genes (DEGs) were identified comparing the livers of PO and pHx groups after batch effect removal (**Figures 4A, B**). In order to define metastasis-associated pathways regulated after pHx, we performed Gene Set Enrichment Analysis (GSEA) with Hallmark gene sets, revealing numerous enriched pathways impacting metastasis (**Figure 4C**), such as the Hallmarks E2F targets and G2M checkpoint. Both gene sets were enriched in the pHx group and encompass important transcripts that regulate cell-cycle progression (**Figure 4D**). In addition to an increased proliferative activity, we observed an enrichment of genes involved in epithelial-mesenchymal transition (**Figure 4C**). The most striking enriched gene sets regarding a potential influence on metastatic seeding were the Hallmark terms angiogenesis, hypoxia, and inflammatory response (**Figures 4E–G**), which are interconnected pathways that potentially mediate the prometastatic effect of the surgical procedure (**Figure 4H**). Therefore, we showed that pHx may facilitate the efficient seeding of LMs due to a prearrangement of prometastatic factors in the liver.

### 3.7 Partial hepatectomy induces genes of the premetastatic niche in residual liver tissue enabling *de novo* metastasis

**Figure 5:**
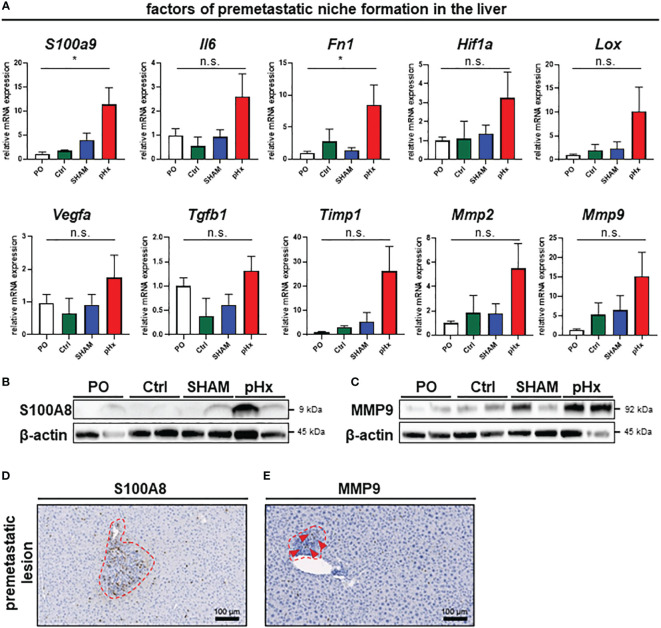
Partial hepatectomy induces premetastatic niche (PMN) formation in the liver. **(A)** Relative mRNA levels (normalized on preoperative values) of the PMN-associated genes *S100a9*, *Il6*, *Fn1*, *Hif1a*, *Lox*, *Vegfa*, *Tgfb1*, *Timp1*, *Mmp2* and *Mmp9* in healthy liver tissue analyzed by qPCR for PO (n=26), Ctrl (n=4), SHAM (n=17) and pHx (n=26) groups; **(B, C)** Western Blot analyses of the expression of the premetastatic niche factors S100A8 and MMP9 from healthy liver (top). β-actin was used as loading control; **(D, E)** Representative IHC stainings of liver sections (bottom) of S100A8 and MMP9, red square marking perivascular premetastatic lesions (scale bars = 100 µm). (Bar plots represent mean ± SEM, p-values calculated via one-way ANOVA Tukey test, * = p<0.05, n.s. non significant.

As we have shown that several genes affecting premetastatic niche formation are increased in the transcriptomic analysis of pHx livers, we analyzed the formation of this special metastasis-favoring niche in pHx treated animals on transcriptomic and proteomic level in depth The residual liver following pHx is defined by an increased abundance of inflammatory cells and increased proliferation. Inflammatory tissue has been described to be more susceptible to metastatic seeding (32) and inflammation is an essential component of the premetastatic niche (10). To further investigate the effects of pHx, we focused on pathways that facilitate engraftment of circulating tumor cells in the organ. Several genes that regulate hepatic hypertrophy after liver resection have also been described in the context of the formation of a premetastatic niche in the liver for metastatic tumors (7, 8, 10). By analyzing the transcriptional profile of the healthy liver tissue via qPCR, we found a significant induction of several genes associated with the premetastatic niche in the hepatectomy mice compared to preoperative levels, as well as to control mice (e.g., the inflammatory genes *S100A9* or *IL-6*, growth factors such as *VEGF* and *TGF-β* or *MMPs* as markers of extracellular remodeling) (**Figure 5A**). These changes in gene expression are specific for partial hepatectomy and not a general effect of surgical trauma, as they were not observed after SHAM surgery (**Figure 5A**). Several of the described genes can be directly linked to neutrophil invasion, either as they attract neutrophils [e.g. *IL-6* (33) *or TIMP1* (34)], or as a result of neutrophil activity [e.g. *S100A9* (35), *Fibronectin* (36), *LOX* (37), *HIF-1α* (38) *or MMP2/9* (39)]. Transcriptional regulation was confirmed at protein level via western blot, where we could also find an upregulation of representative proteins involved in the premetastatic niche (e.g., S100A8 (interaction partner of S100A9, together forming the heterodimer calprotectin) and MMP9) (**Figures 5B, C**). Furthermore, the intrahepatic localization of MMP9 and S100A8 was detected by IHC of liver sections, showing an increased presence of both proteins around perivascular premetastatic lesions (**Figures 5D, E**). Hence, we conclude that prometastatic transcriptional changes along with the inflammatory cellular composition occur in defined sectors within the macroscopically healthy liver and lead together to the formation of premetastatic niches in the liver.

### 3.8 pHx induces tight junction formation in the liver which may impact tumor cell seeding

**Figure 6:**
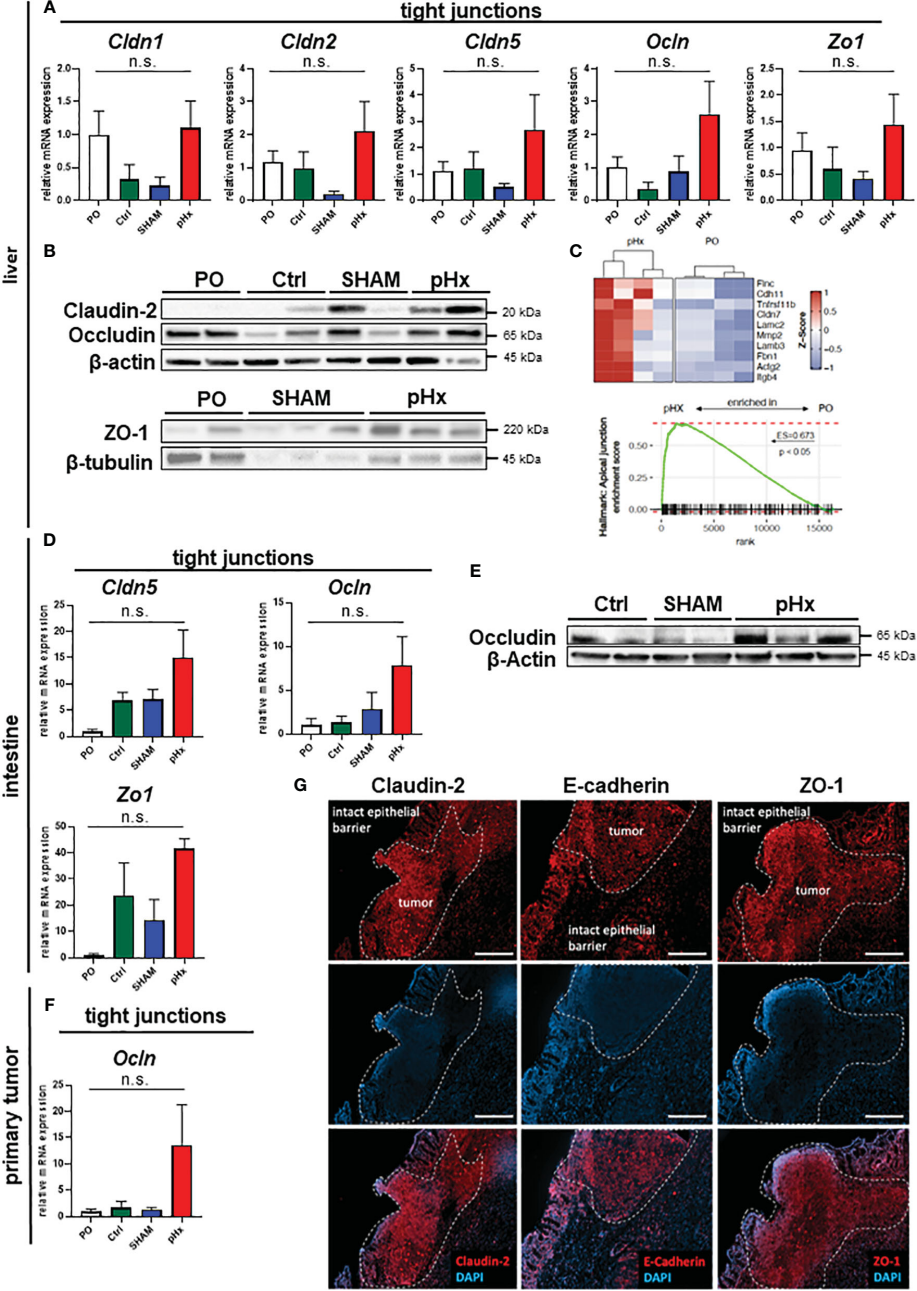
pHx induces tight junction formation in the liver which may impact tumor cell seeding. **(A)** Relative expression of tight junction (TJ) genes *Cldn1*, *Cldn2*, *Cldn5*, *Ocln* and *Zo1* in healthy liver analyzed with qPCR for PO (n=17), Ctrl (n=4), SHAM (n=12) and pHx (n=20) groups; **(B)** Western Blot of selected tight junction proteins Claudin2, Occludin and ZO-1 in the liver. β-actin and β-tubulin were used as loading control; **(C)** Gene Set Enrichment Analysis of bulk RNA sequencing from healthy livers for the hallmark apical junction with heatmap of the 10 leading genes involved according to Z-scores; **(D)** Relative gene expression of *Cldn5*, *Ocln* and *Zo1* in healthy cecum analyzed with qPCR for PO (n=4), Ctrl (n=4), SHAM (n=4) and pHx (n=6) groups; **(E)** Representative Western Blot analysis of protein expression of Occludin in the cecum. β-actin was used as loading control; **(F)** Relative expression of *Ocln* in primary tumors analyzed with qPCR for PO (n=9), Ctrl (n=4), SHAM (n=7) and pHx (n=17) groups; **(G)** Immunofluorescence stainings of tight junction proteins (Claudin-2, E-cadherin, and ZO-1) in healthy epithelium and primary CRC (scale bar = 100 µm). Counterstaining of the nuclei was performed with DAPI. The white dashed lines mark the border between intact and benign epithelial cells and the primary tumors. (Bar plots represent mean ± SEM, n.s. non significant).

One effect of hepatectomy during liver regeneration is an increased formation of tight junctions (TJ) (40). As some members of the zonula occludens proteins are thought to enable intrahepatic extravasation of circulating tumor cells, we analyzed the expression of the genes *Claudin-2* and *-5* as well as *Occludin* and *ZO-1* in liver tissues in this mouse model. We found that *Claudin-2, Claudin-5, Occludin* and *ZO-1* gene expression was increased following pHx compared to PO, Ctrl or SHAM samples in qPCR (**Figure 6A**) and on proteomic level the expression of the tight junction proteins Claudin-2, Occludin und ZO-1 was elevated following pHx in western blot (**Figure 6B**). This finding was supported by RNA sequencing data that showed enrichment of the apical junction gene set (**Figure 6C**). Additionally, we analyzed the expression of several TJ genes in primary tumors and healthy intestines, as they are linked to a worsened prognosis in several tumors and have been shown to drive colorectal carcinogenesis and EMT (41). We found that a similar upregulation of Claudin-5, Occludin and ZO-1 was observed in the healthy intestine both on transcriptomic and proteomic level (**Figures 6D, E**). Furthermore, an upregulation of *Occludin* in primary tumors was detected (**Figure 6F**). As tight junctions are formed in the interactions of several cell types, we stained proteins using immunofluorescence of sections of primary tumors and attached healthy ceca for intestinal localization. In the healthy intestine, TJ proteins Claudin-2, E-cadherin and ZO-1 were located at the membrane of epithelial cells with emphasis on the luminal side of the crypts (**Figure 6G**). In tumor tissue, we found TJ proteins with no spatial orientation and partially within the cytoplasm of the cells, indicating depolarization of the cells (**Figure 6G**), in accordance with the literature.

### 3.9 *De novo* metastasis in pHx could be primarily attributed to premetastatic niche formation in the liver as pHx has no impact on epithelial-mesenchymal transition in primary tumors

Since we observed an increase in *de novo* metastases in our model, we were curious if pHx has an impact on the metastatic potential in the primary tumor. While we detected a trend towards increased *Ki67*-expression in primary tumors (**Supplementary Figures 3A, B**), the expression of genes involved in EMT revealed no significant changes in the expression of *Ctnnb1, Vimentin, Snai1* and *2* or *Zeb1* (**Supplementary Figure 3C**). Additionally, the expression of EMT genes was measured in liver metastases. The expression levels of *Ctnnb1, Snai1* and *2* or *Cdh1* were not influenced by pHx (**Supplementary Figure 3D**).

In summary, we have demonstrated that pHx causes various changes in the cellular and transcriptional composition of the residual liver in a mouse model of orthograde colorectal liver metastasis that can be recapitulated as inflammation, extracellular remodeling, and tight junction formation. The transformation in the residual liver favors further metastatic seeding, while the impact on the primary tumor seems to be insignificant, and ultimately results in an elevated metastatic burden following pHx (see Graphical Abstract).

## 4 Discussion

In this study we present a novel immuno-competent model for studying orthograde CRC liver metastasis and the influence of surgery on tumor progression. Several studies have described the potential negative effects of surgery on different tumors. Tsuchiya et al. found an acceleration of metastatic growth of intravenously injected CRC cells after surgery (13) and Harun et al. found an increased growth of liver metastasis following partial hepatectomy after splenic injection of CRC cells (14). In a study by Brandt et al. (12) tumor cells were directly injected into the remaining liver following different hepatectomies, and they described increased metastatic load depending on the extent of resection. All studies were based on tumor cell injection, excluding important features of the full path of regular *in vivo* metastatic disease. Solid tumors are characterized not only by the primary malignant cells, but especially in CRC the tumor microenvironment and microbiome have a major impact on tumor progression and metastasis. Therefore, an injection model of tumor cells cannot mimic the course of disease in humans. Studying orthotopic metastatic CRC in rodents is difficult because most models do not develop metastasis or are limited by luminal obstruction of the primary tumor. The combination of subserosal organoid implantation into the cecal wall with the aggressive mutational features of the APTAK-organoids offers the possibility to study the influence of pHx in a model of orthograde metastasis, together with immunological effects that highly resemble the human situation in CRC patients. On molecular level the APTAK tumors resemble the CMS4 subtype of human CRC patients (16).

Assessment of the intrahepatic tumor load *in* and *ex vivo* is difficult as the macroscopically visible lesions only represent a small part of the overall metastases and histological analyses usually do not include the entire organ. MRI offers the unique possibility of objectifying the metastatic volume in this model (as even small liver metastases are easily distinguishable in imaging) *in vivo* and at several time points. Yet, microscopic metastases cannot be detected in the MRI. In contrast to previously published results, resection of the medial liver lobe did not accelerate the growth of each metastasis, but rather increased *de novo* metastasis, suggesting priming of the residual liver for metastatic seeding.

The concept of the premetastatic niche (PMN) has attracted the attention of oncological research in recent decades. Following the seed and soil hypothesis of tumor metastasis, priming of the target organ by the tumor is regarded as a promising therapeutic approach for preventing metastasis. The niche is defined by extracellular remodeling via MMPs, inflammation with accumulation of neutrophils, macrophages, regulatory T cells, and bone-marrow derived cells (BMDCs), reprogramming of cancer-associated fibroblasts, and increased angiogenesis (10). Niche formation is induced by several tumor-derived secreted factors that overlap with important regulators of liver regeneration following major hepatectomy (9). The here presented descriptive data strongly suggests that this overlap promotes further metastasis following partial hepatectomy. These premetastatic niches can be detected as inflammatory spots within the liver, dominated by neutrophil and lymphocyte accumulation, and characterized by an increased turnover of the extracellular matrix, without the presence of (malignant) epithelial cells. The central effect of pHx on the residual liver appears to be increased inflammation, as many of the regulated PMN genes are either the cause or effect of the inflammatory phenotype. The extent of the inflammatory response to hepatectomy was also recently described to define successful or failed liver regeneration following hepatectomy (42) and therefore plays a crucial role in short- and long-term results of colorectal LM resection.

We also showed that this response is accompanied by an overall increase in bacterial presence following pHx, which might sustain the inflammation itself. Moreover, hepatic inflammation is not an early, but rather a delayed response, that occurs several days after surgery. Hence, a measurable effect on the metastatic burden was only detected in the MRIs performed on POD14.

Furthermore, liver regeneration is characterized by increased expression of tight junction genes, which help to establish a new hepatic structure (40). Several TJ proteins can be associated with poorer prognosis of cancer patients or accelerated metastasis (41, 43). TJs are thought to mediate direct cell-cell-interactions between hepatocytes and circulating tumor cells, easing the engraftment of new metastases in the liver, but have not yet been described as regulators of the PMN. We found an induction of TJ formation in the healthy liver following pHx, which might attribute to the increase in *de novo* metastases in our model, and could be added to the concept of the PMN. Astonishingly, we also demonstrated that upregulation of TJ proteins can also be observed in the healthy intestine. In the intestine, TJ proteins are involved in colorectal carcinogenesis as regulators and targets of important EMT cascades (e.g., Wnt- or Notch-signaling) and in regulating the cell cycle, inflammation, invasion, and metastasis. CRC itself is characterized by an overexpression of different Claudin genes and by loss of polarization with translocation of TJ proteins to the cytoplasm, which can be seen in the immunofluorescence of primary tumors (44). Transcriptional changes in the intestine also emphasize the crucial effect of hepatectomy on distant organs.

Following metastatic growth in this model, we found that the premetastatic niche is defined by accumulation of neutrophils and both CD4^+^ and CD8^+^ T lymphocytes. Although nearly all immunocytes were excluded during metastatic growth independent of the procedure, indicating immune evasion of the metastasis, we noted a tendency towards reduced T cell count, both for CD8^+^ and CD4^+^ T cells in FACS analyses of livers and LMs after pHx. T cell abundance and function in primary tumors are important for tumor progression and patient prognosis, as indicated by the prognostic value of the immunoscore for different cancer entities (45). Especially cytotoxic CD8^+^ T cells appear to define tumor progression in patients, but their effect has so far only been described in primary tumors and not in metastatic sites. During tumor progression or chronic infection, T cell function is weakened, leading to T cell exhaustion (46). This is thought to be one of the crucial features for successful checkpoint-inhibition in anti-tumor therapies. It has been shown that in several cancers that are usually responsive to PD-1-inhibition, the presence of liver metastasis aggravates the effect of such treatment (47, 48). Therefore, we were curious about the effect of pHx on T cell function. We observed an overall increase in the expression of exhaustion markers on CD8^+^ T cells during tumor progression, consistent with previous murine and human data, but no significant effect of hepatic resection on T cell exhaustion. Our results indicate a possible negative effect of pHx on metastatic CRC in the absence of systemic treatment. As most Stage IV-patients receive systemic therapy prior to the resection of LM, the validation of our findings in humans is difficult, as chemotherapy may prevent or delay intravasation of tumor cells, as well as priming of a premetastatic niche, for example by VEGF-inhibition. These therapeutic modulations should be studied in the future. The clinical benefit of resecting colorectal LM is undoubted, and the combination of modern systemic therapies and more aggressive surgical procedures in UICC Stage IV CRC has achieved significant improvements in the prognosis of patients in the last decades. Nevertheless, a recurrence rate of >50% needs to be addressed in order to reduce the burden of repetitive hepatectomies for patients. Targeting the premetastatic niche in the context of liver surgery appears promising at first sight, but most of the involved pathways are also indispensable for liver regeneration following pHx. Our descriptive data call for a closely timed systemic therapy around pHx (especially in the context of a liver-first approach), to prevent novel metastasis in the vulnerable regenerative period.

In summary, we present a murine model of orthograde CRC metastasis in which pHx causes increased *de novo* metastasis and a generally higher intrahepatic tumor burden by priming a premetastatic niche dominated by inflammation, angiogenesis, and overexpression of tight junctions in the residual liver.

## Data availability statement

The datasets presented in this study can be found in online repositories. The names of the repository/repositories and accession number(s) can be found below: https://www.ncbi.nlm.nih.gov/geo/, GSE253251.

## Ethics statement

The animal study was approved by Regierungspräsidium Freiburg, Freiburg, Germany. The study was conducted in accordance with the local legislation and institutional requirements.

## Author contributions

JL: Conceptualization, Data curation, Formal analysis, Investigation, Methodology, Project administration, Validation, Visualization, Writing – original draft, Writing – review & editing. FH: Data curation, Formal analysis, Investigation, Validation, Writing – original draft, Writing – review & editing. RF: Data curation, Formal analysis, Investigation, Methodology, Supervision, Writing – original draft, Writing – review & editing. BM: Data curation, Formal analysis, Investigation, Methodology, Supervision, Writing – original draft, Writing – review & editing. CB: Conceptualization, Funding acquisition, Investigation, Methodology, Resources, Supervision, Writing – original draft, Writing – review & editing. JR: Data curation, Formal analysis, Investigation, Methodology, Visualization, Writing – original draft, Writing – review & editing. DP: Formal analysis, Investigation, Methodology, Writing – original draft, Writing – review & editing, Conceptualization, Data curation. LM: Conceptualization, Data curation, Formal analysis, Investigation, Methodology, Supervision, Writing – original draft, Writing – review & editing. WR: Conceptualization, Data curation, Formal analysis, Funding acquisition, Investigation, Methodology, Project administration, Resources, Supervision, Visualization, Writing – original draft, Writing – review & editing. DV: Conceptualization, Funding acquisition, Investigation, Methodology, Project administration, Resources, Supervision, Writing – original draft, Writing – review & editing. DR: Conceptualization, Methodology, Project administration, Supervision, Visualization, Writing – original draft, Writing – review & editing. CL: Conceptualization, Formal analysis, Investigation, Methodology, Supervision, Visualization, Writing – original draft, Writing – review & editing. AJ: Conceptualization, Formal analysis, Investigation, Methodology, Supervision, Visualization, Writing – original draft, Writing – review & editing. HN: Conceptualization, Formal analysis, Investigation, Methodology, Project administration, Supervision, Visualization, Writing – original draft, Writing – review & editing. PH: Conceptualization, Formal analysis, Investigation, Methodology, Project administration, Supervision, Visualization, Writing – original draft, Writing – review & editing. SF-F: Conceptualization, Funding acquisition, Investigation, Methodology, Project administration, Resources, Supervision, Visualization, Writing – original draft, Writing – review & editing. RK: Conceptualization, Data curation, Formal analysis, Funding acquisition, Investigation, Methodology, Project administration, Resources, Supervision, Validation, Visualization, Writing – original draft, Writing – review & editing.
