# Supplementary material for: Partial hepatectomy accelerates colorectal metastasis by priming an inflammatory premetastatic niche in the liver
Source: Front Immunol. 2024 Jun 10;15:1388272. doi: 10.3389/fimmu.2024.1388272 (PMC11196966; doi:10.3389/fimmu.2024.1388272)
Supplement: Supplementary file 1 [file Presentation_1.pdf]

Supplementary Figures

Supplementary Figure 1:

(A) Representative IHC staining of EpCam in premetastatic lesions (red circles), liver metastasis and primary tumor (scale bar = 100 µm); **(B)** Image of the surgical set-up before and after resection of the medial hepatic lobe; **(C)** Volume of healthy liver in right and left lobes on postoperative day (POD) 14 of PO group (n=18), Ctrl group (n=6), SHAM group (n=6) and pHx group (n=6); **(D)** Time course analysis of total healthy liver volume in µl of Ctrl group (n=6), SHAM group (n=6) and pHx group (n=9) ; **(E - F)** Total volume (E) and number (F) of LM in MRI in right and left lobes on POD14 of Ctrl group (n=7), SHAM group (n=7) and pHx group (n=9); **(G)** Time course analysis of combined volume of LM in all lobes in µl of Ctrl group (n=7), SHAM group (n=15) and pHx group (n=19)

(Bar plots represent mean ± SEM, p-values calculated via one-way ANOVA Tukey test, * = $p<0.05$; ** = $p<0.01$; *** = $p<0.001$))

Supplementary Figure 2:

(A) Flow cytometric (FACS) analysis of innate immune cells (neutrophils and monocytes) in LM (top) and peripheral blood (bottom) of PO group (n=5), Ctrl group (n=3), SHAM group (n=6/6) and pHx group (n=12/12); **(B)** FACS analysis of myeloid-derived suppressor cells (MDSC) in the liver (top) with changes in CD14-expression in PMN-MDSCs (bottom) of PO group (n=4), Ctrl group (n=6), SHAM group (n=5) and pHx group (n=8); **(C)** concentration (ng/µl) of bacterial DNA in LM in quantitative real-time polymerase chain reaction (qPCR) of Ctrl group (n=3), SHAM group (n=3) and pHx group (n=3); **(D)** FACS analysis of adaptive immune cells ($CD3^+$ T cells, $CD4^+$ T cells, $CD8^+$ T cells, B cells and NK cells) in LM; **(E)** Flow cytometric analysis of exhaustion, cytotoxicity and activation markers (granzyme B, T-bet, Tcf1, PD1, CD38) in $CD8^+$ T cells in healthy liver of PO group (n=3), Ctrl group (n=6), SHAM group (n=6) and pHx group (n=8).

M-MDSC=monocytic MDSC, PMN-MDSC=polymorphonuclear MDSC

(Bar plots represent mean ± SEM)

Supplementary Figure 3:

(A) Representative IHC staining of Ki67 in primary tumor in the orthotopic organoid transplantation mouse model (scale bar = 200 µm); **(B)** *Ki67*-gene expression in primary tumors analyzed by qPCR; **(C)** Expression of epithelial-mesenchymal transition (EMT)-genes (*Epcam*, *Ctnnb1*, *Cdh1*, *Snai1*, *Snai2*, *Zeb1* and *Vim*) in primary tumors (analyzed by qPCR) of PO group (n=9), Ctrl group (n=4), SHAM group (n=9) and pHx group (n=21); **(D)** EMT-gene expression (*Epcam*, *Ctnnb1*, *Cdh1*, *Snai1*, *Snai2*) in LM analyzed by qPCR of PO group (n=3), SHAM group (n=6) and pHx group (n=9).

(Bar plots represent mean ± SEM)

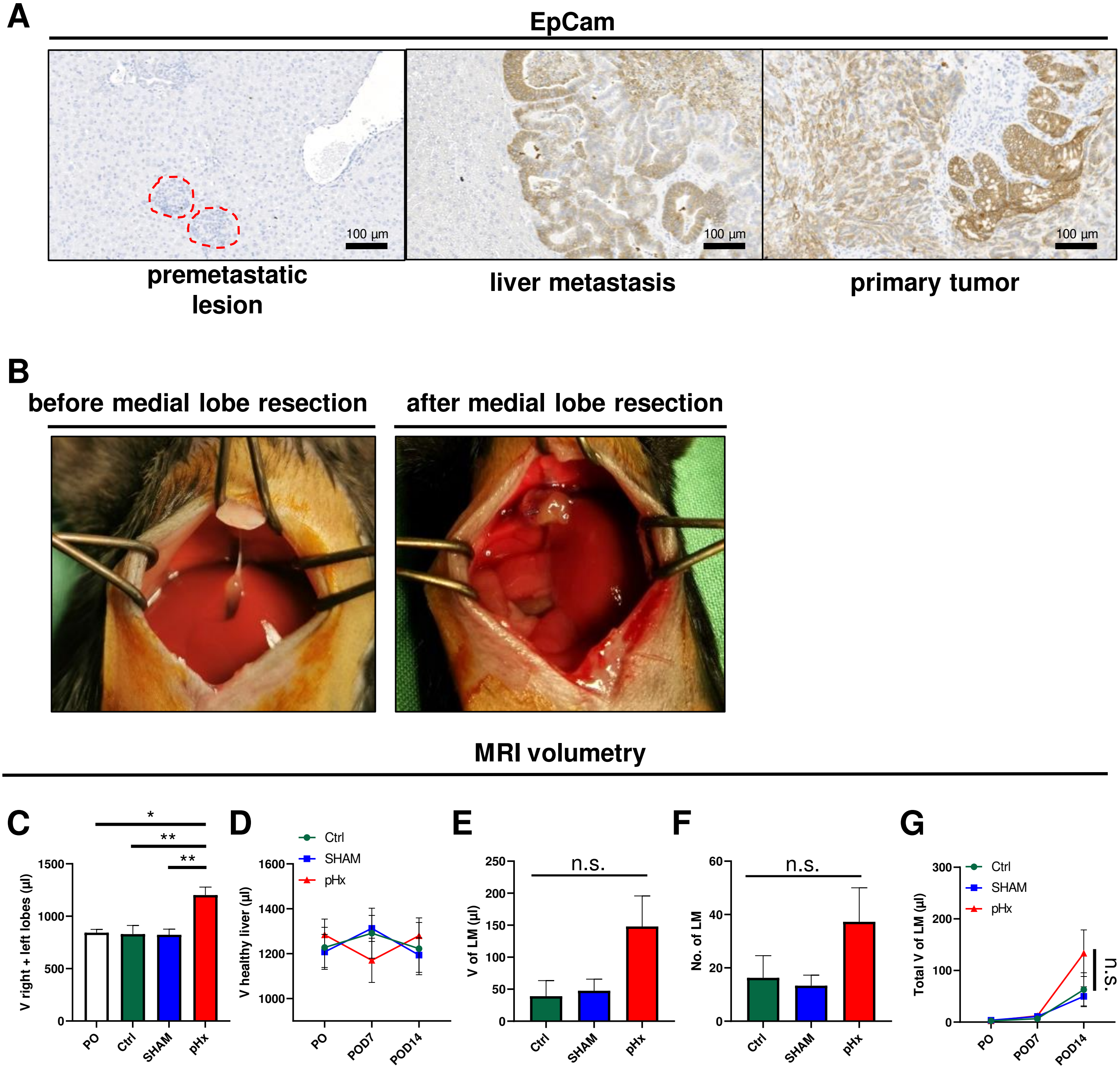

Supplementary Figure 1

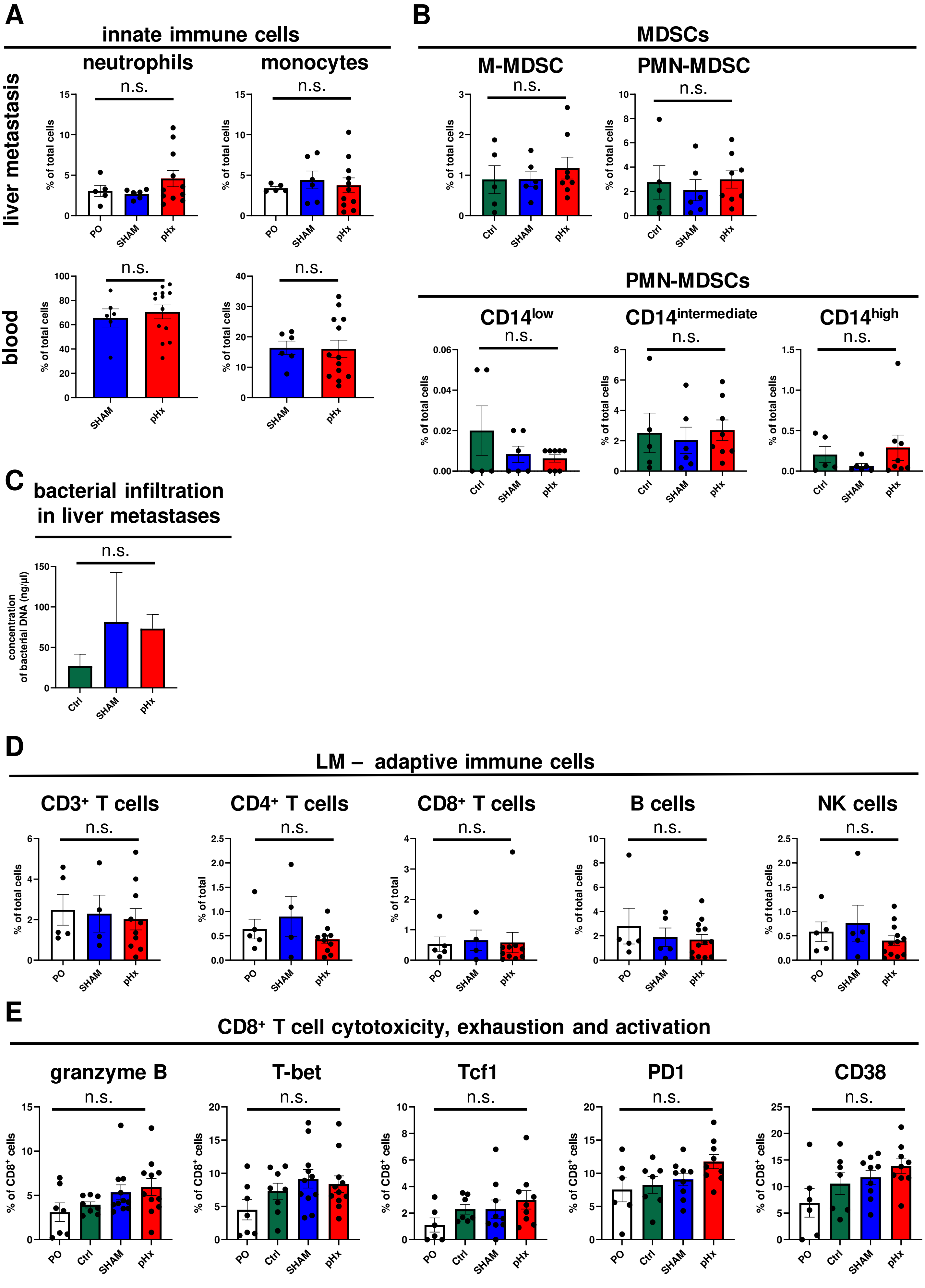

Supplementary Figure 2

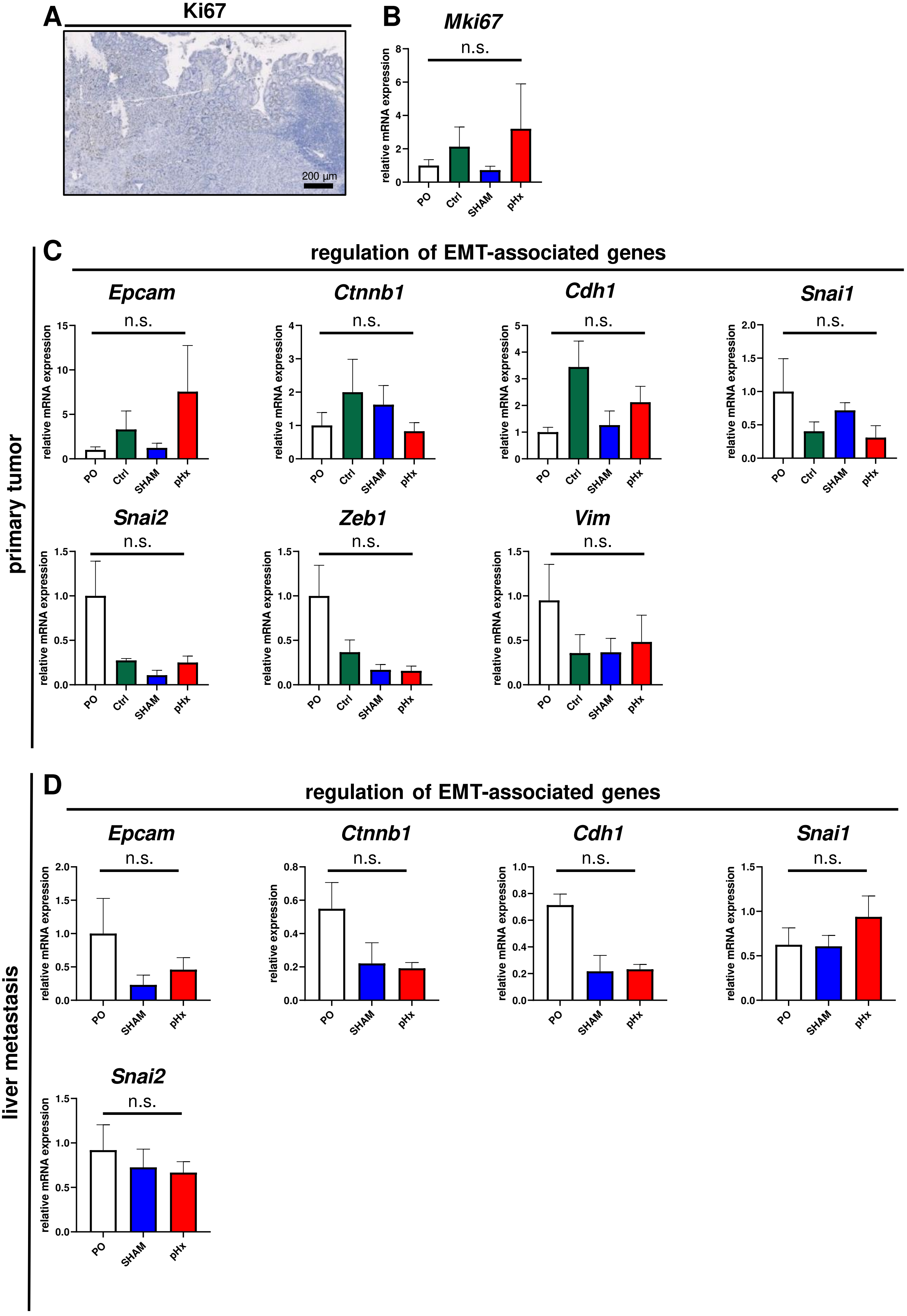

Supplementary Figure 3

Supplementary Methods

Antibodies - Immunohistochemistry/Immunofluorescence

**Antibody**	**Supplier**	**Isoform**	**Catalogue-Nr.**	**Dilution**
Ki67	Cell Signaling	D3B5	#9129	1:300
CD4	Cell Signaling	D7D2Z	#25229	1:200
CD8	Invitrogen	4SM16	#14-0195-82	1:25
S100A8	Cell Signaling	E4F8V	#47310	1:1000
MMP9	Abcam	EPR22140-154	ab228402	1:1000
Claudin-2	Cell Signaling	E1H9O	#48120	1:50
Occludin	Cell Signaling	E6B4R	#91131	1:50
ZO-1	Cell Signaling	D6L1E	#13663	1:50
EpCam	Cell Signaling	VU1D9	#2929	1:500
DAKOenVisionK4003 (Anti rabbit)	Agilent DAKO	K4003	K400311-2	1:250
Rat on mouse HRP	Zytomed		RT517G	Ready to use
donkey antiRabbit IgG AF568	Invitrogen	A10042	# A10042	1:500

Antibodies - Western Blot

**Antibody**	**Supplier**	**Isoform**	**Catalogue-Nr.**	**Dilution**
β-Actin	Cell Signalling	13E5	#4970	1:1000
S100A8	Cell Signaling	E4F8V	#47310	1:1000
MMP9	Abcam	EPR22140-154	ab228402	1:1000
Claudin-2	Cell Signaling	E1H90	#48120	1:1000
Occludin	Cell Signaling	E6B4R	#91131	1:1000
ZO-1	Cell Signaling	D6L1E	#13663	1:1000
rabbit-IgG-HPR	Cell Signaling		#7074	1:2000

Antibodies - Fluorescence activated cell sorting (FACS)

Antibody	Supplier	Isoform	Catalogue-Nr.	Dilution
B220 - APC	BioLegend	RA3-6B2	103211	undiluted
CD3 – BV421	BioLegend	17A2	100227	undiluted
CD3 – BV785	BioLegend	17A2	100231	undiluted
CD4 – PercpCy5.5	BioLegend	GK1.5	100434	undiluted
CD8a – PE-Dazzle594	BioLegend	53-6.7	100761	undiluted
CD11b - PeCy7	BioLegend	M1/70	101215	undiluted
CD11c - APC	BD Biosciences	HL3	561119	undiluted
CD14 - PE	BioLegend	Sa14-2	123309	undiluted
CD16/32	BioLegend	93	101319	undiluted
CD19 - FITC	BioLegend	1D3/CD19	152404	undiluted
CD38 - PE	BioLegend	90	102707	undiluted
CD45 - e450	Thermo Fisher Scientific	30-F11	48-0451-80	undiluted
CD45 – AF700	BioLegend	30-F11	103127	undiluted
CD64 - PercpCy5.5	BioLegend	X54-5/7.1	139307	undiluted
FoxP3 - PE	BioLegend	150D	320012	undiluted
GranzymeB – PE Cy7	BioLegend	QA16A02	372213	undiluted
IL-10 – Vio515	Miltenyi	REA1008	130-116-968	undiluted
Ly6G - FITC	BD Biosciences	1A8	551460	undiluted
Ly6C - PerCPCy5.5	BioLegend	HK1.4	128012	undiluted
MHCII - PE	BioLegend	M5/114.15.2	107608	undiluted
NK1.1 – BV510	BioLegend	PK136	108737	undiluted
NK1.1 – BV605	BioLegend	PK136	108739	undiluted
PD1 – BV510	BioLegend	29F.1A12	135241	undiluted
Tbet – AF488	BioLegend	4B10	644829	undiluted
Tcf1 – AF488	R&D Systems	812145	IC8224G	undiluted

qPCR Primers

Gene	5‘-3‘ Sequence forward	5‘-3‘ Sequence reverse
18S	GTAACCCGTTGAACCCCATT	CCATCCAATCGGTAGTAGCG
Cldn2	AAGGCCTAGGATGTAGCCCA	CGGCTCCGTTTTCTAGATGC
Cldn5	TTCTATGATCCGACGGTGCC	ACTTGACCGGGAAGCTGAAC
Cdhn1	ATCCTCGCCCTGCTGATT	ACCACCGTTCTCCTCCGTA
Epcam	GATTCTGCACGTGAGACCTG	GATACCAAGTCAAACCGAGAACTT
Fn1	CGGAGAGAGTGCCCCTACTA	CGATATTGGTGAATCGCAGA
Hif1a	CATGATGGCTCCCTTTTTCA	GTCACCTGGTTGCTGCAATA
Il6	GCTACCAAACTGGATATAATCAGGA	CCAGGTAGCTATGGTACTCCAGAA
mKi67	AGGGTAACTCGTGGAACCAA	TCTTAACTTCTTGGTGCATACAATG
Lox	TATGCCTGCACCTCTCACAC	TGTCCGCATTGTATGTGTCAT
Mmp2	AACTTTGAGAAGGATGGCAAGT	TGCCACCCATGGTAAACAA
Mmp9	AGACGACATAGACGGCATCC	TCGGCTGTGGTTCAGTTGT
Ocln	AGGCACGGGTAGCACTCACG	CATAGTTCTTCTTGTCGTAATC
S100a9	CAGTTGGCAACCTTTATGAAGAA	CTCAGCTGATTGTCCTGGTTT
Snai2	CATTGCCTTGTGTCTGCAAG	CAGTGAGGGCAAGAGAAAGG
Snai1	GTCCAGCTGTAACCATGCC	TGTCACCAGGACAAATGGGG
Tgfb1	TGGAGCAACATGTGGAACTC	GTCAGCAGCCGGTTACCA
Timp1	GCAAAGAGCTTTCTCAAAGACC	AGGGATAGATAAACAGGGAAACACT
Vegfa	TTAAACGAACGTACTTGCAGATG	AGAGGTCTGGTTCCCGAAA
Vimentin	CCAACCTTTTCTTCCCTGAAC	TTGAGTGGGTGTCAACCAGA
Zeb1	ACCCCTTCAAGAACCGCTTT	CAATTGGCCACCACTGCTAA
Zo1	GCCTTGAACTTTGACCTCTGC	CAGAAATCGTGCTGATGTGCC
Ctnnb1	TGCAGATCTTGGACTGGACAT	AAGAACGGTAGCTGGGATCA

Primer Bacterial 16s qPCR

5’-CNACGCGAAGAACCTTANC-3’

5’- ATACGCGARGAACCTTACC-3’

5’-CTAACCGANGAACCTYACC-3’

5’- CAACGCGMARAACCTTACC-3’

5’-CGACRRCCATGCANCACCT-3’

Other items (drugs, equipment, kits etc.)

Item	Supplier
BioSpec 94/21	Bruker Biospin, Germany
Beckman Coulter Gallios	Beckman Coulter, USA
BD LSRFortessa	Becton Dickinson, Switzerland
AxioScan 7	Zeiss, Germany
ChemiDoc XRS+	BioRad, USA

Cultrex RGF basement membrane extract (BME) Type 2	R&D Systems, USA
CollagenI rat tail	Ibidi, Germany
Tumor dissociation kit, mouse	Miltenyi Biotec, Germany
Liver dissociation kit, mouse	Miltenyi Biotec, Germany
RNAeasy mini kit	Qiagen, Netherlands
DNeasy Blood & Tissue Kit	Qiagen, Netherlands
ProtoScript II First Strand cDNA Synthesis Kit	New England Biolabs, USA
QuantiTect SYBR Green RT PCR mastermix	Qiagen, Netherlands
Isofluran-Piramal	Piramal, India
Buprenorphine (Temgesic)	Schering-Plough, USA
Seprafilm	Baxter, USA
Vicryl 6-0	Ethicon, USA
